# Mining of unexplored habitats for novel chitinases—*chiA* as a helper gene proxy in metagenomics

**DOI:** 10.1007/s00253-012-4057-5

**Published:** 2012-04-25

**Authors:** Mariana Silvia Cretoiu, Anna Maria Kielak, Waleed Abu Al-Soud, Søren J. Sørensen, Jan Dirk van Elsas

**Affiliations:** 1 Department of Microbial Ecology, University of Groningen, Nijenborgh 7, 9747 AG Groningen, the Netherlands; 2 Department of Biology, University of Copenhagen, Copenhagen, Denmark

**Keywords:** *chiA*, Bacterial community, Environment, Functional screening, *chiA* pyrosequencing

## Abstract

The main objective of this study was to assess the abundance and diversity of chitin-degrading microbial communities in ten terrestrial and aquatic habitats in order to provide guidance to the subsequent exploration of such environments for novel chitinolytic enzymes. A combined protocol which encompassed (1) classical overall enzymatic assays, (2) *chiA* gene abundance measurement by qPCR, (3) *chiA* gene pyrosequencing, and (4) *chiA* gene-based PCR-DGGE was used. The *chiA* gene pyrosequencing is unprecedented, as it is the first massive parallel sequencing of this gene. The data obtained showed the existence across habitats of core bacterial communities responsible for chitin assimilation irrespective of ecosystem origin. Conversely, there were habitat-specific differences. In addition, a suite of sequences were obtained that are as yet unregistered in the chitinase database. In terms of *chiA* gene abundance and diversity, typical low-abundance/diversity versus high-abundance/diversity habitats was distinguished. From the combined data, we selected chitin-amended agricultural soil, the rhizosphere of the Arctic plant *Oxyria digyna* and the freshwater sponge *Ephydatia fluviatilis* as the most promising habitats for subsequent bioexploration. Thus, the screening strategy used is proposed as a guide for further metagenomics-based exploration of the selected habitats.

**Electronic supplementary material:**

The online version of this article (doi:10.1007/s00253-012-4057-5) contains supplementary material, which is available to authorized users.

## Introduction

Being one of the most abundant biopolymers in nature, chitin (polymer consisting of β-1,4-linked-*N*-acetyl-glucosamine) has been studied in many domains of science, from chemistry and biomedicine to synthetic material development (Agullo et al. 2003; Muzzarelli 2009). The turnover of chitin is dependent on hydrolysis, resulting in small oligosaccharide chains, which may become further accessible to different metabolic and biochemical processes. In soil and marine ecosystems, chitin degradation is a key step in the cycling of both nitrogen and carbon (Gooday 1990). Chitin-degrading enzymes (chitinases) are present in many natural systems and they are widely distributed among all three domains of life (Li and Greene 2010; Gao et al. 2003). They are responsible for hydrolysing the glycosidic bonds of chitin, thus releasing dimeric (chitobiose) and monomeric (*N*-acetylglucosamine, GlcNAc) compounds.

Overall, most relevant chitinases belong to the glycoside hydrolase families 18 and 19. The classification into these two groups is based on amino acid sequence similarities (Henrissat 1991; Henrissat and Davies 2000), substrate specificities and structures of the catalytic domains of the respective enzymes. The family-18 and -19 enzymes have different structures and modes of action. Based on the structure of the catalytic domain and on the position of the hydrolysis site on the polysaccharide chain, chitinases may show either endo- or exoactivity (Henrissat and Davies 2000; Van Scheltinga et al. 1994).

In nature, chitin degradation is likely carried out by complexes of enzymes rather than by single enzymes. The study of the enzymatic complexes involved in the degradation of chitin and chitin derivatives has been spurred by the potential that the (partially) degraded compounds offer (Jayakumar et al. 2011). Recent studies indicate that, although these enzymes have been found in many prokaryotic and eukaryotic organisms, the highest quantity of chitin is turned over by both bacteria and fungi in marine and terrestrial ecosystems (Delpin and Goodman 2009; Poulsen et al. 2008). The carbohydrate enzyme database (CAZy) Cantarel et al. 2009) indicates the existence of more than 2,500 chitin-active enzymes and associated proteins (e.g., chitin-binding proteins) of bacterial origin. Despite this high number, only one group of bacterial chitinases has been extensively characterized so far, i.e. group A chitinases, proposed as being most abundant in nature (Li and Greene 2010; Metcalfe et al. 2002a; Suzuki et al. 1999).

So far, a few studies have assessed bacterial chitinase gene diversities in different terrestrial and aquatic ecosystems (Hjort et al. 2010; Hobel et al. 2005). Also, the biochemical and functional properties (including antibacterial and antifungal activities) of chitinases obtained from specific bacteria isolated from different habitats have been investigated (LeCleir et al. 2004; Metcalfe et al. 2002b; Williamson et al. 2000). By using culture-dependent and -independent methods, considerable diversities of *chiA* genes and predicted catalytic activities were found. Moreover, the effects of environmental factors and of chitin and its oligomers on natural bacterial communities have been addressed (Cottrell et al. 2000; Metcalfe et al. 2002a; Orikoshi et al. 2005).

The ability to degrade chitin appears to be very important in a wide range of bacterial species, whether they have a free-living lifestyle or are associated with biofilms or with different eukaryotes (e.g. symbiotic bacteria). Hence, screening of key habitats with respect to chitinase gene abundance and diversity is a necessary first step for further metagenomics-based exploration and exploitation of these for industrial purposes.

The aim of the present study was therefore to screen a suite of different (terrestrial and aquatic) habitats with respect to their bacterial communities involved in chitin degradation and to assess the natural prevalence of the respective enzymes for further biotechnological applications. Next to direct enzymatic activity measurements (indicating potential overall activity), we addressed the molecular diversity and abundance of bacterial chitin degraders to evaluate the probability of detection of novel chitinases with enhanced features. We hypothesized that in habitats with different origin and characteristics, chitinolysis is driven by dissimilar microbial communities. Thus, we provide information on the ecology of the bacterial communities that are involved in the degradation of chitin, in particular with respect to the pool of *chiA* genes, as a prelude to future metagenomic and functional analyses.

## Materials and methods

### Selection and sampling of habitats

Ten different microbial habitats (six terrestrial and four aquatic ones) were selected for functional and genetic screenings of the chitinolytic bacterial communities. The selection of habitats was based on evidence provided by previous studies on the potential richness of chitinolytic bacterial species and diverse enzymatic activities (Chaston and Goodrich-Blair 2010; Gerce et al. 2011; Hjort et al. 2010; Kennedy et al. 2007; Manucharova et al. 2006; Metcalfe et al. 2002a; Sjoling et al. 2007; Terahara et al. 2009; Williamson et al. 2000).

The terrestrial habitats were represented by agricultural soil either or not following addition of chitin, spent mushroom substrate (SMS), wood-based biofilter material and arctic plant (*Oxyria digyna* and *Diapensia lapponica*) rhizospheres.

The soil samples were collected from an experimental field (Vredepeel, the Netherlands) either treated with chitin (further referred as “soil chitin”, SC) or not (further referred as “soil non-chitin”, SNC). Sampling was done nine months after the (second) treatment with 20 tons of commercial chitin (Gembri, Ecoline Biotechnology, the Netherlands) per hectare. Data of a pilot study (data not shown) recommended this type of soil. The SMS sample consisted of soil-like material, with high organic matter content and was collected after 3 weeks production of *Agaricus bisporus brunensis* (Agarica BV, Hoogeveen, the Netherlands). The biofilter material originated from a wood-based filter (Cyprio BV, Groningen, the Netherlands) used for treatment of gas released from an industrial ethanol production site.

Moreover, the *O. digyna* and *D. lapponica* rhizospheres represented as-yet-unexplored habitats in terms of excreted bacterial enzymes. Both plants are known for their ability to survive at low temperatures, under harsh conditions (temperatures are predominantly below zero and the rhizospheres undergo frequent freeze–thaw cycles). The presence of an active bacterial endophytic community was also reported (Nissinen, submitted for publication). The plants were selected within a 50-m-diameter site (Kilpisjarvi, Saana, Finland) and the rhizosphere was carefully collected from the plant roots.

The samples obtained from all terrestrial environments comprised three different sampling plots (for soil, wood-filter), plants (rizhosphere), shelves (spent mushroom compost) different amount of material. Different characteristics like pH, water and organic content were determined (Table S1A and B). Four different species of sponges—one freshwater and three marine—were investigated. The freshwater sponge *Ephydatia fluviatilis* was sampled from a shipwreck at about 7 m depth (Vinkeveense Plassen, the Netherlands). The marine species *Halichondria panicea* (Easter Schelde Estuary, the Netherlands), *Corticium candelabrum* and *Petrosia ficiformis* (Santa Anna, Blanes, Spain) are common sponges living on hard substrata between 5 and 45 m of depth. From each sponge, between 2.5 and 5 g of wet tissue was sampled and placed in 50-ml tubes filled with local water. Further on, in the laboratory all samples were washed and brushed three times under sterile distilled water to remove adhering hard material and the thin external biofilm.

Irrespective of their origin, three replicates were used for all samples analyzed in the present study. All enzymatic and genetic analyses were performed for all three replicates of each sample.

### Chitinase activity

Enzymatic screening was based on detection of the (relative) activity of exo-chitinases (β-*N*-acetylglucosaminidase and chitibiosidase). Fresh material (0.5 g) was suspended in 1.2 ml sterile distilled water and the mixture was homogenized (5 min), followed by centrifugation at 12,000 × *g* for 5 min (based on the SMS sample requirements). The supernatant (10 μl) was assayed using 4-methylumbelliferyl-*N*-acetyl-β-d-glucosaminide (substrate for β-*N*-acetylglucosaminidase) and 4-methylumbelliferyl *N*,*N*-diacetyl-β-d-chitobioside (substrate for chitobiosidase). The protocol was followed as described in the chitinase assay kit instructions (Sigma, Saint Louis, USA). One unit of chitinase activity was considered as the release of 1 μmol of 4-methylumbelliferone from the appropriate substrate per min at pH 5.0 at 37 °C.

### DNA isolation

DNA was isolated from all samples using the PowerSoil kit (MoBio Laboratories, Inc., Carlsbad, CA, USA). Isolation was performed from 500 mg fresh material by following the manufacturer’s instructions. DNA concentration and purity were measured using a NanoDrop apparatus (ThermoFisher Scientific, St. Leon-Rot, Germany) and confirmed by gel electrophoresis (comparing with fragments of a 1-kb DNA ladder; Fermentas, ThermoFisher Scientific, St. Leon-Rot, Germany).

### PCR-DGGE analysis of bacterial 16S *rRNA* and *chiA* genes

Table 1 summarizes the primers, thermal profiles and DGGE conditions used to analyze the communities investigated in this study. Both the *chiA* and 16S rRNA genes were assessed via a nested PCR approach. All PCRs were carried out in 50-μl volumes containing 5 μl of 10× PCR buffer, 0.2 mM dNTPs mix, 3.75 mM MgCl_2_, 2 % DMSO, 20 μm of each primer, and 1.5 U GoTaq Flexi DNA polymerase (Promega, Madison, USA). All amplifications were performed on a Veriti 96-well thermal cycler (Applied Biosystems, Life Technologies Europe BV, Bleiswijk, the Netherlands).

**Table 1:** List of oligonucleotides and PCR conditions used in this paper

Target	Primers	PCR protocol	Analysis	Reference
Bacterial 16S rRNA gene	pA/1492R followed by 968FGC/1378R; Eub338/Eub518	55 °C; 35 cycles; touchdown 65 to 55 °C; 35 cycles; 65 °C; 40 cycles	DGGE (40–60 % denaturant; QPCR	Edwards et al. 1989; Heuer et al. 1997; Fierer et al. 2005
Chitinase A (*chiA*)	GA1F/ GA1R followed by GASQF/GASQR	55 °C; 30 cycles	DGGE (40–50 % denaturant)	Williamson et al. 2000
	GA1F/ GA1R	55 °C; 40 cycles	QPCR	Yergeau et al. 2007

The amplification conditions were preceded by an initial denaturation step (95 °C for 5 min) and followed by a final elongation step (72 °C for 10 min). For each cycle of PCR denaturation was at 94 °C for 1 min, annealing at the specific temperature (Table 1) for 1 min and elongation at 72 °C for 1 min. Ten nanograms of DNA was used as template in PCR reactions.

DGGE was performed using an Ingeny PhorU2 system (Ingeny Phor U2, Goes, the Netherlands), as described in the manufacturer’s instructions. In order to have equal amount of PCR product (100 ng), different volumes were loaded onto 8 % (*w*/*v*) polyacrylamide gels (40 % acrylamide/bis 37,5:1; BioRad) with gene-specific denaturing gradient (100 % denaturant is defined as 7 M urea and 40 %, *v*/*v*, formamide; Muyzer and Smalla 1998). Separation of fragments was performed in TAE buffer (40 mM Tri-acetate, 20 mM sodium acetate, 1 mM EDTA, pH 8.0) at 60 °C, 15 min at 200 V, followed by 75 V for an additional 16 h. The gel was stained with SYBR Gold nucleic acid gel stain (Invitrogen) according with manufacturer specifications. The similarities of the densitometric curves of the patterns were calculated using the Pearson correlation coefficient (GelCompar fingerprint and gel analysis software, Applied Maths, Sint-Martens, Latem, Belgium). There were no major differences observed among the replicates per sample type. Thus, for cluster analysis, one replicate per sample type was used.

### Quantitative PCR based on 16S rRNA and *chiA* genes

Quantification of the 16S rRNA and *chiA* genes was performed using a Maxima SYBR Green system (Fermentas, ThermoFisher Scientific, St. Leon-Rot, Germany) on an Applied Biosystems 7300 Real-Time PCR System (Applied Biosystems, Life Technologies Europe BV, Bleiswijk, the Netherlands). Primer sets are shown in Table 1. PCR conditions were established in accordance with instructions by the fluorochrome detection system manufacturer. An eight-point standard curve was constructed from tenfold dilutions corresponding to 10^1^ to 10^8^ copies of *chiA* (approximately 450 bp) and full-length 16S rRNA gene products obtained after amplification of pure template DNA isolated from a *Streptomyces griseus* chitinase producing strain.

### Pyrosequencing of the chiA catalytic domain

Specific sequencing adaptors (tags) were designed and attached to PCR primers originally used for detection of *chiA* (Table 1). In order to increase the amount of possible template, a double round PCR approach was performed. Purified specific pooled PCR products (10 ng) generated with non-sequencing primers were used as the template for the second PCR before sequencing. PCR mixes and conditions were similar with those used for PCR-DGGE-based screening. Pyrosequencing reactions were carried out on a GS FLX pyrosequencing system according to the manufacturer’s instructions (Roche Diagnostics GmbH/454, Life Science Corporation, Brandfort, CT, USA).

### Sequence analysis

Mothur (http://www.mothur.org/) was utilized to analyze the pyrosequencing reads. The obtained sequences were evaluated for quality, ambiguous bases, and homopolymer content. All sequences which did not pass quality control requirements (meaning quality reads above 25, no ambiguous bases, homopolymers below 10 bases), as well as sequences without identifiable primers and barcode, were removed. Furthermore, sequences were trimmed to remove primers and barcodes. Sequences which did not align correctly were also removed from the dataset. Potential chimeras were removed using Chimera Slayer implemented in Mothur. The remaining reads were translated into amino acid sequences. All sequences containing internal stop codons and unidentified amino acids due to sequencing errors were then removed. Qualified sequences were assigned to operational taxonomic units (OTUs) based on a 20 % dissimilarity cut-off.

### Statistical analyses

Non-transformed data were used for generating the graphical distribution of the investigated functions. Furthermore, the enzymatic assay data were integrated with the qPCR results and analyzed in Statistica version 8.0 (StatSoft, GBH, Germany). Data normality was tested with the Shapiro–Wilk’s test. An appropriate transformation was applied (log and square root transformation) for the data which were considered to have failed one of the tests.

## Results

### Chitinase activities across habitats

**Fig. 1:**
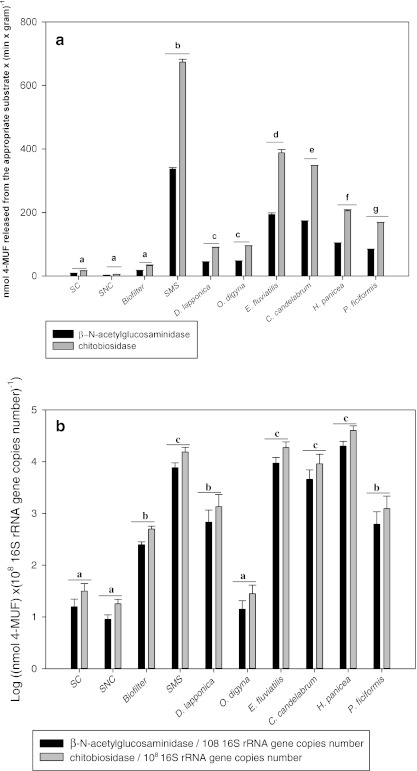
**a** Estimated chitinase activity expressed as 4-methylumbelliferone released from 4-methylumbelliferyl *N*-acetyl-β-d-glucosaminide and 4-methylumbelliferyl *N*,*N*′-diacetyl-β-d-chitobioside/min/g fresh material. **b** Estimated exochitinase activity expressed as 4-methylumbelliferone released from 4-methylumbelliferyl N-acetyl-β-d-glucosaminide and 4-methylumbelliferyl *N*,*N*′-diacetyl-β-d-chitobioside/min normalized over 16S rRNA gene copy numbers. Values that are statistically different among habitats (*p* < 0.0001) are indicated

The chitin-degrading activities present in each habitat were measured using crude cell suspensions (containing 10^5^ to 10^8^ bacterial cells/ml) prepared from the samples. First, chitobiosidase activity was found to be dominant for each sample type. Detailed analysis of the data revealed seven patterns of exochitinase activities (Fig. 1a). Among the soil and soil-like habitats, three activity levels were detected. The lowest activity level was found in SNC, an intermediate one in SC and a high level in the biofilter. No differences in activities were measured across the Arctic rhizosphere samples. Finally, the four sponge samples revealed quite divergent activity levels. The highest one was detected in *E. fluviatilis* and the lowest in *P. ficiformis*. Compared to all other samples, the SMS sample showed significantly raised activity levels (*p* < 0.0001). The exochitinase activities measured were then normalized over the 16S rRNA gene copy numbers (see next section). On the basis of this normalization, there appeared to be three patterns of activities across the different habitats. Thus, the normalization showed differences among arctic rizhosphere and similar activities in fresh-water and marine sponges (Fig. 1b).

### Abundance of bacterial communities, including those with chitin degrading capacity, across habitats

To provide a basis to the divergent chitinase activity levels across habitats (singling out the bacterial contribution), direct molecular analyses of the microbiota in term of abundance (real-time PCR) and composition (PCR-DGGE) were the next steps applied. This analysis thus pinpoints possible differences across the bacterial communities that are potentially involved in chitin degradation. Therefore, microbial community DNA was successfully extracted and purified from all sampled habitats.

**Fig. 2:**
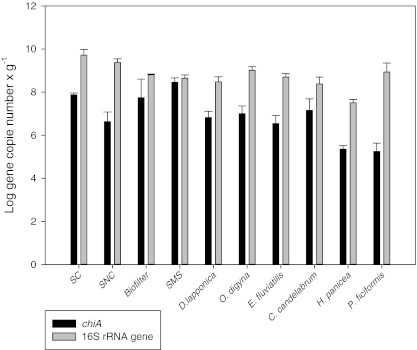
Real-time quantification of 16S rRNA and *chiA* genes across ten different habitats (per gram of fresh material)

The 16S rRNA gene copy numbers determined by real-time PCR were taken as a proxy for bacterial abundances in the samples (Fig. 2). The abundances (sizes of the bacterial communities per unit mass) showed low variation across the samples, from 10^7^–10^8^ gene copies/g tissue in sponges and in SMS to 10^9^/g soil and in the *O. digyna* rhizosphere. In fact, the SC sample showed significantly higher 16S rRNA gene abundances than most of the other samples (Table S2A). Furthermore, the abundance of the 16S rRNA gene in the *H. panicea* was the lowest and significantly different from that in the other habitats, except in *D. lapponica*, *E. fluviatilis*, and *C. candelabrum*.

The *chiA* gene copy numbers showed variations, ranging from 10^5^ to 10^8^ gene copy numbers/g of material. The lowest copy numbers were found in the *P. ficiformis, E. fluviatilis* and *H. panicea* samples, while the SC, biofilter and SMS samples tended to have higher abundances (Fig. 2). The abundance of *chiA* in SC, biofilter and SMS was significantly different from that in the sponges, except in *C. candelabrum* (Table S2B). Also, the *chiA* gene copy numbers were then used as a proxy for the size of the family-18 bacterial chitin-degrading communities (Fig. S1).

### PCR-DGGE-based analysis of the diversity of bacterial communities, including those with chitin-degrading capacity, across habitats

#### Phylogenetic diversity

**Fig. 3:**
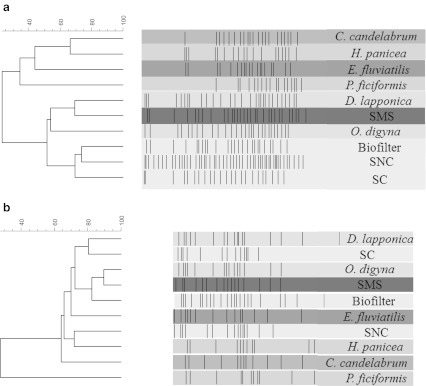
Clustering of PCR-DGGE profiles based on UPGMA and the Pearson correlation coefficient. *Solid blocks* represent the gradient of enzymatic activity (high color intensity corresponds to high enzymatic activity). **a** 16S rRNA gene-based PCR-DGGE profiles showing the clustering of the bacterial communities in soil-like and sponge samples. **b** *chiA* gene-based PCR-DGGE profiles showing clustering of *chiA* gene pools. Note the similar structure in approx. 70 % of selected habitats

All habitats samples yielded 16S rRNA gene based PCR products of the expected sizes, as evidenced from gel electrophoresis (data not shown). These mixed PCR products were then subjected to DGGE fingerprinting, yielding bacterial community fingerprints for all samples. Overall, the analyses showed great consistency across replicates of the same sample. Moreover, diverse bacterial communities were found to dwell in all habitats, as judged from the average band numbers as well as their position. These numbers varied from 17 in the marine sponge *P. ficiformis* to 52 in SNC (Fig. 3a). Cluster analysis (UPGMA; Pearson coefficient) revealed the existence of two broad clusters, separated by the habitat type (terrestrial versus aquatic). These two clusters came together at 20 % similarity. Among the soil/soil-like samples, at 51 % similarity, two subgroups were distinguishable. One subgroup was formed by the Arctic plant rhizosphere samples together with SMS, and the other one by the biofilter and SNC and SC samples.

#### ChiA gene diversity

All environmental DNAs yielded *chiA*-gene based amplicons which were separable on DGGE gels. Detailed analyses of the resulting DGGE banding patterns revealed a rather limited *chiA* gene richness. Specifically, the numbers of bands varied from 11 (in the sponges *H. panicea* and *P. ficiformis*) to 23 (in the biofilter).

Cluster analysis revealed that, over all habitats sampled, the *chiA* gene based PCR-DGGE profiles were similar to each other at 63 % of similarity (Fig. 3b). Thus, the profiles of all habitats, except of the marine sponge *P. ficiformis,* grouped closely together. From this observation, the existence of a core *chiA* gene community which is similar across all habitats may be proposed (Fig. 3b). Remarkably, the *chiA* gene profiles from the rhizosphere of *O. digyna* grouped with those of SMS (90 % similarity). Furthermore, at 83 % similarity those from the *D. lapponica* rhizosphere clustered with those from SC.

### Deep sequencing analysis of *chiA* gene diversity across habitats

We performed *chiA* gene based direct pyrosequencing of the selected environmental samples. In total, 172,804 uncurated sequences were obtained. After removal of chimeric sequences and/or those of poor quality, a total of 40,105 robust sequences were obtained for all samples. The highest number of sequences was obtained for *E. fluviatilis* (9,341) and the lowest for the biofilter (4,053) samples. The estimated numbers of *chiA* gene types, based on a 20 % difference criterion, varied from 40 (in the biofilter) to 308 (in SNC). Using the 20 % criterion for binning of the sequences, we then performed rarefaction analysis, allowing a calculation of the Chao1 and ACE *chiA* gene richness estimators (Fig. S2). This analysis revealed still increasing trends for the majority of the communities. Only for *E. fluviatilis*, a tendency to reach a horizontal plateau was found (Fig. S2). Both the Chao 1 and the ACE estimators pinpointed SNC as the habitat with the highest estimated richness values (1,240 and 2,115, respectively; Table 2). The lowest estimates of richness (53 and 58) were estimated for the biofilter. On the basis of the data, we then calculated the richness and evenness of *chiA* gene types per habitat. The resulting Shannon diversity indices varied from a minimum of 1.1 (in the biofilter) to a maximum of 3.3 (in SNC). The comparative analyses of the reads from all samples, including the numbers of sequences, numbers of OTUs, estimated richness, diversity indices and coverage estimators, are shown in Table 2.

**Table 2:** Comparison of *chiA* gene pyrosequencing reads for all samples

Sample	No. sequences	No. types	ACE	*Chao1*	Shannon	Coverage
SNC	7157	308	2115	1240	3.3	0.93
SC	5318	119	305	222	2.4	0.98
SMS	7291	131	1025	487	2.5	0.97
Biofilter	4053	40	58	53	1.1	0.99
*Ephydatia fluviatilis*	9341	56	251	205	1.3	0.99
*Oxyria digyna*	4810	139	335	221	2.5	0.97
*Diapensia lapponica*	2135	109	245	182	2.3	0.98

Including number of sequences, number of types, estimated richness and diversity indexes, and Good’s coverage estimator

**Fig. 4:**
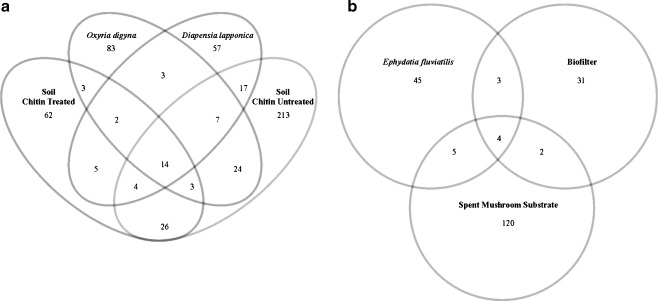
Venn diagrams showing the uniqueness versus sharedness of *chiA* gene sequences between samples. **a** Shared OTUs among SNC, SC, arctic rhizospheres; **b** shared OTUs among SMS, the biofilter and *Ephydatia fluviatilis*

We then analyzed the numbers of unique and shared *chiA* gene types among the habitats. The resulting Venn diagrams clearly show that defined numbers of OTUs were actually shared between the samples whereas others were not (Fig. 4), pointing at both considerable uniqueness of some of the habitats, next to some commonality, in terms of *chiA* gene types present.

### Tentative association of *chiA* gene types with those from defined bacterial taxa

All *chiA* gene sequences were used as queries for BLAST-P analyses against the database, which also contained 1,754 bacterial glucoside hydrolase family-18 protein sequences from CAZy (Cantarel et al. 2009). The query sequences were assigned to reference sequences according to the top BLAST hits (Table S3). In all samples, sequences with best hits to those from *Actinobacter*ia, *Bacteroidetes*, *Chloroflexi*, *Deinococcus-Thermus*, *Dictyglomi*, *Firmicutes*, and *Proteobacteria* were identified (Fig. S3). The sequences thus attributed to the *Proteobacteria* revealed best hits with diverse sequences from the *Beta*-, *Gamma*-, and *Deltaproteobacteria* classes. Sequences affiliated with those of *Actinobacteria* were dominantly found (65 % to 98 % of all sequences) across all habitats, with SC and *E. fluviatilis* being the only habitats where actinobacterial *chiA* sequences were not the major group. Thus, *Proteobacteria-*, and in particular *Betaproteobacteria-*like sequences, were most abundant in SC (53 %) and in *E. fluviatilis* (98 %; Fig. S4). Among all identified phyla, *Dictyglomi-*like *s*equences were found only in SNC.

**Fig. 5:**
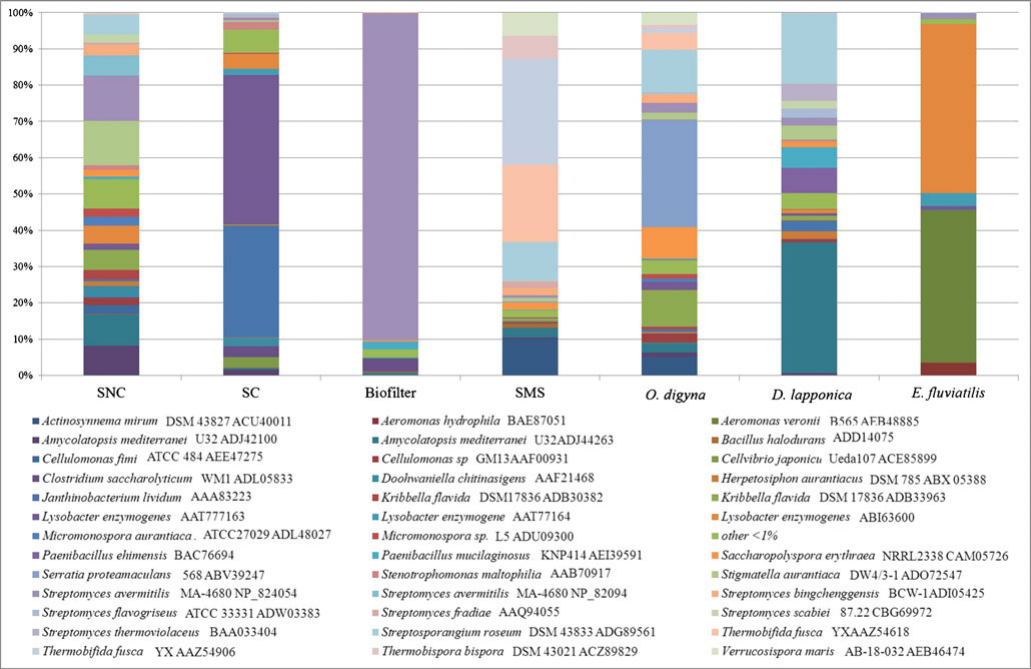
Distribution of *chiA* sequence species types in the habitats

When the distribution of *chiA* sequence types was considered at the species level (Fig. 5), a few sequence types appeared as conspicuously most abundant in several habitats. In SNC, sequences affiliated with those from *Amycolatopsis mediterranei* U32 (accession number ADJ42100), *A. mediterranei* U32 (ADJ44263), *Stigmatella aurantiaca* DW4/3-1 (ADO72547), *Streptomyces avermitilis* MA-4680 (NP-824054) and *Streptosporangium roseum* DSM 43021 (ACZ89829) appeared. In contrast, sequences similar to those of *Janthinobacterium lividum* (AAA83223) and *Lysobacter enzymogenes* (AAT77163) were found to be dominant in SC. In the biofilter sample, 90 % of the sequences were similar to a sequence of *S. avermitilis* MA 4680 (NP824054). Two sequence types, namely one of *Thermobifida fusca* YX (AAZ54618 and AAZ54906), and one each of *S. roseum* DSM 43021 (ACZ89829) and *Actinosynnema mirum* DSM 43827 (ACU40011) were found in SMS. The arctic rhizosphere samples showed similar compositions in terms of dominant *chiA* types, with differences in abundances. A *chiA* gene sequence affiliated with one of *Serratia proteamaculans* 568 (ABV39247) was most abundant in *O. digyna* and one of *A. mediterranei* U32 (ADJ44263) in *D. lapponica*. The *chiA* gene related to *S. roseum* DSM 43021 (ACZ89829) was found in both samples. The freshwater sponge *E. fluviatilis* revealed a unique pattern, with *chiA* sequences affiliated with those of *Aeromonas veronii* B565 (AEB48885) and *Lysobacter enzymogenes* (ABI63600) covering the majority of reads.

## Discussion

In this study, we screened ten different habitats for a highly diverse bacterial chitinolytic function. The aim of the study, i.e., the assessment of defined aspects of chitinolysis across habitats, was based on the premise that a better understanding of the ecology of chitinolysis will serve our subsequent exploration of habitats on the basis of educated guesses as to the occurrence of chitinases with unexplored features. We thus applied parallel function- and gene-based methodologies to evaluate the habitat specificity of family-18 chitinase genes using a combination of proxies for *chiA*-specific abundances and diversities compared with total bacterial ones.

By testing, using crude cell extracts, the total relative exochitinase (β-*N*-acetylglucosaminidase and chitobiosidase) activities across habitats, we found that, expectedly, this function is expressed at different levels under the conditions applied. For all samples, the chitobiosidase activities were higher than the β-*N*-acetylglucosaminidase ones, however this difference may be artifactual, as the measured activities were based on the degradation of chitin oligomers and not on true chitin. There may also have been overlapping activities due to method limitations. The substrate of chitobiosidase (4-methylumbelliferyl *N*,*N*-diacetyl-β-d-chitobioside) can be also cleaved, at lower efficiency, by β-*N*-acetylglucosaminidase, thus the activity measured can be the result of the combined activity of both enzymes. The activities showed a significant raise in SMS, which may have come about as a result of the presence of fungal decomposition material (Krsek and Wellington 2001; Poulsen et al. 2008). Moreover, the high activity in sponges, at least in the marine ones, may relate to the chitinous material they commonly ingest with the (sea) water that is filtered. For reasons unknown, the activities in both SNC and SC were low. It is possible that the chitin-degrading enzyme systems which were present in the soils actually did not get induced to a sufficient extent under the experimental conditions applied, which is possibly due to the presence of easier nutrient sources in the soil. As it is known that the *chiA* gene is abundant among different radiations of the *Bacteria*, real-time PCR, in conjunction with PCR-DGGE, was used to evaluate the abundances, diversities and structures of the *chiA* gene pools across the ten habitats sampled. To normalize the data, we also determined the bacterial community sizes on the basis of the 16S rRNA gene pools. Concerning this, with two exceptions, rather expected bacterial abundances were found. The highest *chiA* gene abundance was observed in the *O. digyna* rhizosphere, and the lowest one in the marine sponge *H. panicea*. No correlation with sampling site characteristics, plant growth stage (flowering or winter form) or host organism-related factors could be shown. The increase of the bacterial community size after chitin addition to soil was consistent with earlier reports (Hallmann et al. 1999; and Metcalfe et al. 2002a). Among the sponge samples, no clear differences were observed between the freshwater and marine sponges. The species investigated by us may have up to 40 % of their biomass represented by metabolically active bacteria (Taylor et al. 2007).

Remarkably, on the basis of the degenerated primers of Williamson et al. (2000), *chiA* was identified in the bacterial communities in all environments. The *chiA* gene copy numbers differed among habitats, both as absolute and as normalized (i.e., per bacterium) numbers. Although *chiA* gene abundance cannot be taken as a proxy for the actual bacterial enzymatic activity (given potential other limiting factors as argued in the foregoing), the normalized abundance provides information about the chitinolytic potential, in terms of the prevalence of a target gene, of the bacterial communities. The finding that the SMS sample had the highest *chiA* gene abundance, followed by SC and the biofilter, whereas the Arctic rhizospheres were similar to the SNC sample, is consistent with the assumed roles of bacterial chitinolysis in these habitats. Remarkably, the high score for SMS in this respect coincided with a high normalized enzyme activity found for this habitat. Such correlations were also found for the SC and SNC soils, the biofilter and the Arctic rhizospheres. However, they were erratic for the sponges, where the highest normalized *chiA* abundance was found in *C. candelabrum* and the highest normalized enzyme activity in *H. panicea*.

To characterize the bacterial communities, we determined their structure at the level of 16S rRNA gene based PCR-DGGE and *chiA* gene composition (using pyrosequencing). The phylogenetically based DGGE profiles divided all habitats in two main groups in accordance with their (terrestrial or aquatic) origin. As expected, the terrestrial samples showed the highest diversity of the dominant populations, as judged by the numbers (richness) and relative intensities (evenness) of bands. A decrease of diversity in the Vredepeel soil was observed as a result of the chitin treatment, as over 50 % of the bands of the SNC (control) were not visible in the SC samples. We assume this to be an effect of growth stimulation of a limited subset of chitinolytic bacteria resulting from the added chitin. The lack of a clear clustering of the DGGE profiles among the sponges might indicate commonality of bacterial types across these.

In terms of *chiA* gene-based diversity detected by PCR-DGGE, each habitat appeared to have a habitat-specific genetic pool of *chiA* genes. Next to this apparent habitat specificity, a core *chiA* gene pool (which was shared across most communities) became visible on the basis of an analysis of occupied band positions. Thus, although the existence of unique, habitat-specific bands yielded evidence for the contention that any selected habitat would yield unique hits with potential for biotechnological investigation, also commonality in terms of chitinase types will be found there.

A comparison of the *chiA*-based DGGE profiles with the bacterial ones revealed that the former had lower diversity in all habitats. So far, only few studies have addressed the relationship between the *chiA* and 16S rRNA gene based phylogenetic distributions (Cottrell et al. 2000; Metcalfe et al. 2002a; Ramaiah et al. 2000). Although the present study does not propose to analyze the relation between the numbers and diversities of *chiA* and 16S rRNA genes, this is an important facet which supports our understanding of both potential in situ chitinolysis and possibilities for the metagenomics mining of the underlying enzymes.

Finally, as part of the deep genetic evaluation of all habitats, the bacterial family-18 chitinase pool was examined by direct pyrosequencing. To our knowledge, this is the first study that uses tag-based amplicon pyrosequencing to study the diversity of the *chiA* catalytic domain across habitats. Pyrosequencing allows the identification of potential chitinases. Such enzymes are recorded in CAZy and related enzyme databases (e.g., BRENDA, EXPASY) with characterized functional parameters. In addition, the analysis allowed the presumptive detection of chitinase producers on the basis of gene sequences but not based on functional parameters of active enzymes.

The SC sample was dominated by two types of *chiA* sequences that resembled those of *Beta*- (*J. lividum)* and *Gammaproteobacteria* (*L. enzymogenes).* A significant decrease of the *chiA* gene diversity in soil after the chitin treatment was observed, which is consistent with previous reports (in which actinobacterial chitinases were dominant) (Krsek and Wellington 2001; Metcalfe et al. 2002a). However, in the present study, the total share of *Actinobacteria* was less than 30 %. We explain this difference with previous work by taking into account the time of sampling, i.e. 9 months after the chitin treatment, which differed from that used in the previous studies (Krsek and Wellington 2001; Metcalfe et al. 2002a). Gammaproteobacterial-like *chiA* genes were also found to dominate in the *E. fluviatilis* bacterial communities (i.e., affiliated with species like *A. veronii* and *L. enzymogenes*). Considering the aim of this study, the two species, *J. lividum* and *L. enzymogenes*, will become interesting targets for mining of novel chitinases. *J. lividum* is known as secreting at least two exochitinases (Gleave et al. 1995), while for *L. enzymogenes* completely characterized chitinases A are as-yet unavailable.

The data provided by both the functional and genetic analyses in this study reinforce the hypothesis that habitat-specific ecological factors are driving the structures of local chitinolytic communities, and, by this, the mode and rate of chitin degradation. However, our focus was on the bacterial contribution to this process and hence fungal involvement remained unexplored. Given the plausible assumption that, in addition to fungal, different bacterial enzyme complexes are involved in the process, this gives credit to the contention that a careful choice of the (biased or unbiased) habitat for sampling will enable a directed metagenomics-based access to desired target genes. Moreover, the tools developed and used by us allow an improved assessment and prediction of hit rates in any subsequent metagenomics exploration of selected habitats, which in this study are the chitin-treated soil SC, the rhizosphere of the Arctic plant *Oxyria digyna* and the freshwater sponge *Ephydatia fluviatilis*. Thus, a guidance strategy for metagenomics mining can be designed on the basis of the developed techniques, the application of which is essential for success, as the rate of positive hits of interesting genes is likely to be greatly raised by careful examination of the data.

## Electronic supplementary material

Below is the link to the electronic supplementary material.

ESM 1 (DOCX 580 kb)
