# Supplementary material for: Mining of unexplored habitats for novel chitinases—chiA as a helper gene proxy in metagenomics
Source: Appl Microbiol Biotechnol. 2012 Apr 25;94(5):1347–58. doi: 10.1007/s00253-012-4057-5 (PMC3353111; doi:10.1007/s00253-012-4057-5)
Supplement: Supplementary file 1 — (DOCX 580 kb) [file 253_2012_4057_MOESM1_ESM.docx]

**Mining of unexplored habitats for novel chitinases – *chiA* as a helper gene proxy in metagenomics**

Mariana Silvia Cretoiu^a^, Anna Maria Kielak^a^, Waleed Abu Al-Soud^b^, Søren J. Sørensen^b^ , Jan Dirk van Elsas^a^

**^a^**Department of Microbial Ecology, University of Groningen, Nijenborgh 7, 9747 AG, Groningen, The Netherlands

^b^Department of Biology, University of Copenhagen, Copenhagen, Denmark

**Corresponding author:** Mariana Silvia Cretoiu

Tel.: +31(0)503632161, Fax: +31(0)503632412; email: [m.s.cretoiu@rug.nl](mailto:m.s.cretoiu@rug.nl)

Nijenborgh 7, 9747 AG, Groningen, The Netherlands

**Supplementary material**

**Figures legends**

Fig. S1. Relative abundance of *chiA* gene copy numbers

Fig. S2. Rarefaction curves of the average number of OTUs within each of the environmental samples. Sequences were classified into OTUs based on the 20% dissimilarity cut off

Fig. S3. Distribution of total bacterial phyla (*chiA* pyrosequencing data from all habitats included) identified

Fig. S4. Distribution of bacterial phyla across habitats

Fig.S1


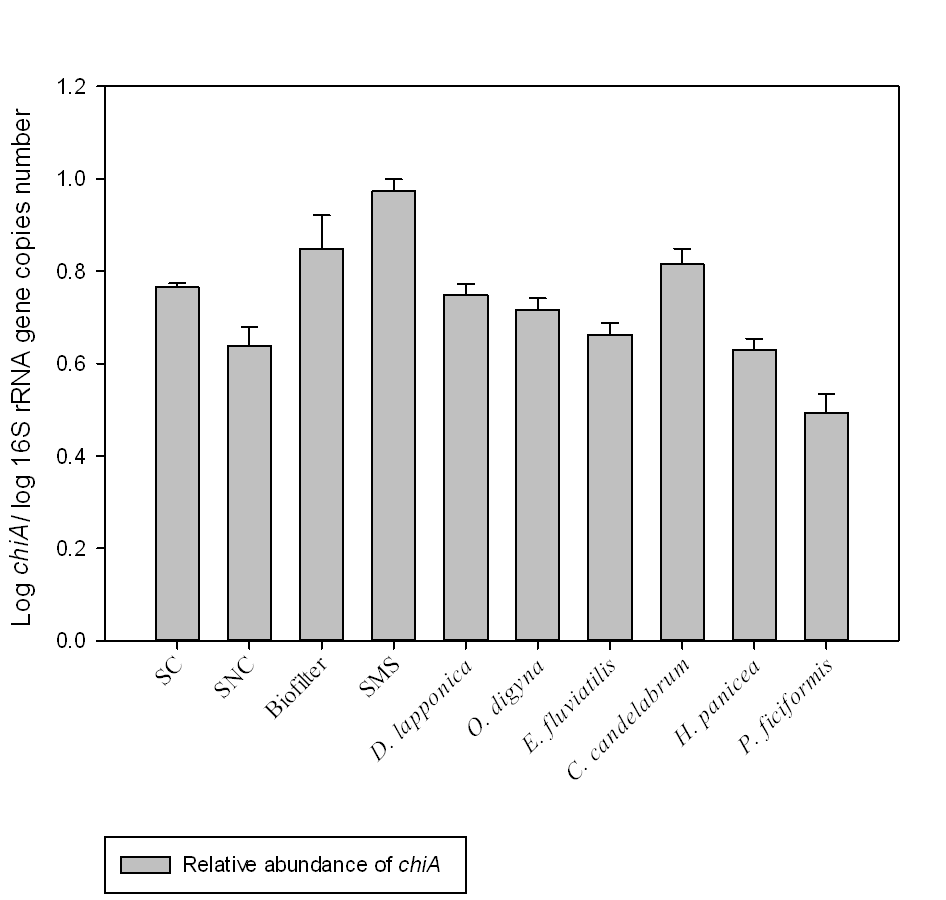


Fig.S2


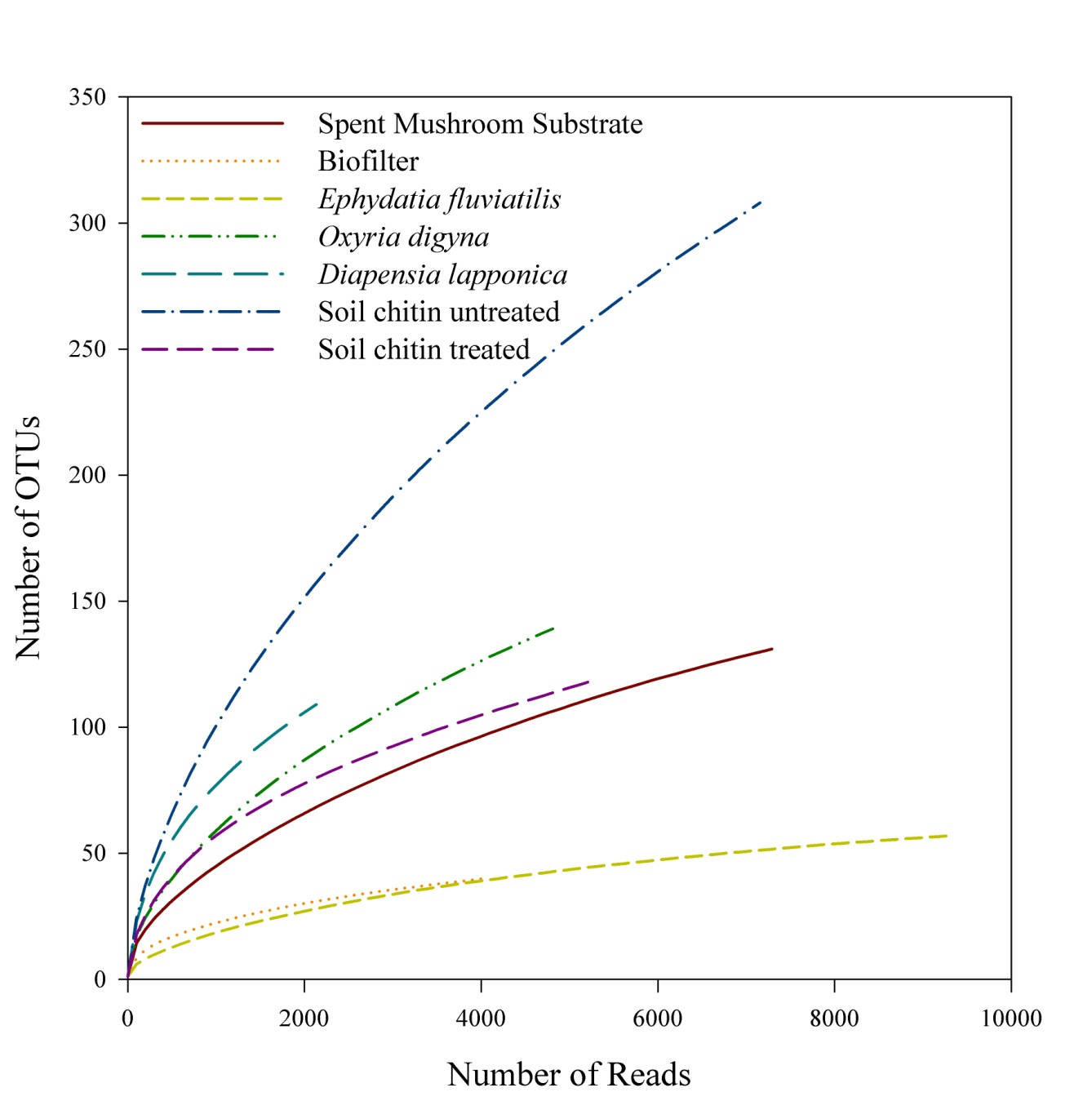


Fig.S3

**
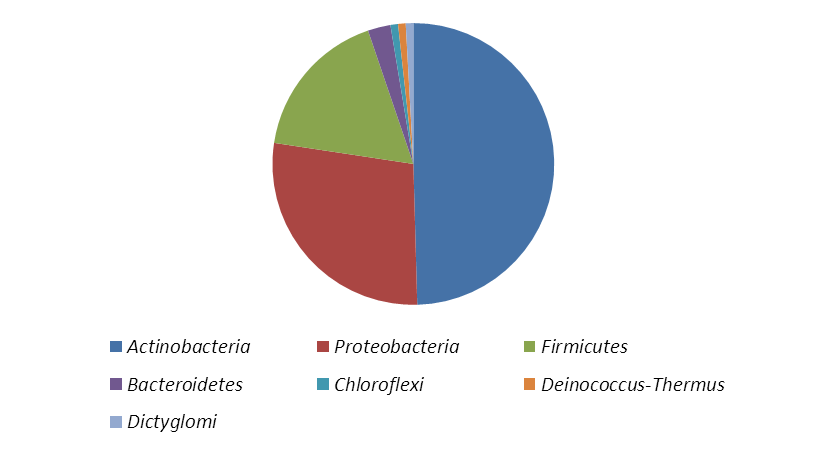
**

Fig.S4


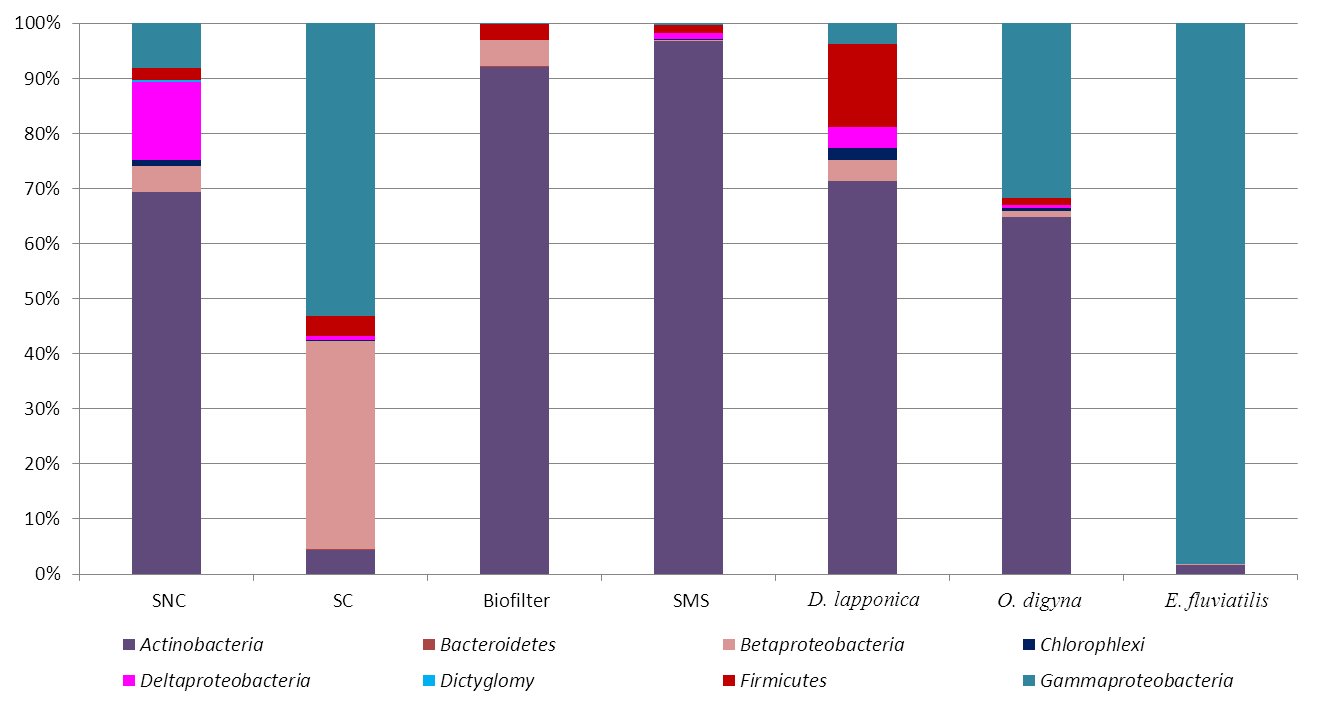


**Table S1.** Characteristics of samples used in the study

**A.**

*na – data not available

**B.**

**Table S2** Relative abundance of 16S rRNA gene (A) and *chiA* (B) copy numbers – significant differences among habitats are indicated (P <0.05)

**A.**

**B.**

**Table S3** Average of identity (%) between the most abundant best BLASTP hits and sequences obtained in this study
